# Development and application of the TFA macrosimulation model: a case study of modelling the impact of trans fatty acid (TFA) elimination policies in Brazil

**DOI:** 10.1186/s12889-022-14361-9

**Published:** 2022-11-02

**Authors:** Eduardo Augusto Fernandes Nilson, Neha Khandpur, Fabio da Silva Gomes

**Affiliations:** 1 Center for Epidemiological Research in Nutrition and Public Health, University of São Paulo, São Paulo, Brazil; 2 Pan American Health Organization, Washington, USA

**Keywords:** Trans fatty acids, Cardiovascular disease, Cardiovascular disease, Health economics, Food policy, Public health, Global health

## Abstract

**Introduction:**

The consumption of trans-fatty acids (TFA) is directly associated with cardiovascular disease risk and is responsible for a significant health burden globally. The policy strategies for reducing TFA include limiting their content in foods and eliminating partially hydrogenated oils (PHO) in the market. This study aims to describe a comparative risk assessment macrosimulation model and to apply this tool to estimate the potential reductions in CVD mortality gained from the compared scenarios of TFA reduction/elimination in Brazil.

**Methodology:**

We developed and implemented a comparative risk assessment macrosimulation model estimates the potential CVD mortality reduction (coronary heart disease – CHD- and stroke) if TFA intake is reduced in diets. The TFA macrosimulation model estimates the change in the annual number of NCD deaths between baseline with current TFA consumption levels and alternate or counterfactual scenarios, such as considering different limits to TFA content in foods and the elimination of PHO in Brazil in 2018. The model incorporated additional outputs related to other impacts of TFA reduction on DPP, such as Years of Life Lost, Years of Productive Life Lost, and related economic impacts of premature deaths.

**Results:**

In 2018, a 2% limit for TFA in the oils and fats and a 5% limit of TFAs for other foods could avert or postpone approximately 2,000 deaths (UI 95% 1,899-2,142) and save US$ 32.1 million savings in productivity losses to the economy associated to premature deaths. An intermediate scenario, applying a 2% limit of TFA in all food products In Brazil could prevent or postpone approximately 6,300 deaths (UI 95% 5,925-6,684) and the premature deaths prevented would represent US$ 100.2 million in economic saving. Finally, by banning PHO, approximately 10,500 deaths could be prevented or postponed (UI 95% 9,963 − 10,909), corresponding to US$ 166.7 million in savings to the economy because of premature deaths.

**Conclusion:**

The TFA macrosimulation model can efficiently compare different policy scenarios for trans fats reduction policies at the country level and proves that the elimination of PHOs from the food market in Brazil may significantly reduce the health burden of trans fatty acids in the country compared to other policy options. The model also represents a useful public health tool to support TFA reduction and elimination policies in other countries.

**Supplementary Information:**

The online version contains supplementary material available at 10.1186/s12889-022-14361-9.

## Introduction

Noncommunicable diseases (NCDs) are the leading cause of global mortality globally, affecting more people each year than all other causes combined and dietary risk factors represent the largest burden among the modifiable NCD risk factors [1]. According to the 2019 Global Burden of Disease Study, non-communicable diseases (NCDs) account for 73.4% (72.5–74.3%) of global mortality, or 42.0 million (40,1–43,9) deaths and 1,62 billion (1.43–1.81) Disability-Adjusted Life Years (DALYs) [2]. Premature mortality from NCDs results in over 15 million deaths of people between the ages of 30 and 69 years, more than 85% of whom live in low- and middle-income countries [3].

In 2019, cardiovascular diseases (CVD) such as ischemic heart disease and stroke, the most common causes of NCD mortality, were responsible for an estimated 15.8 million deaths and 349.5 million DALYs [4]. In some regions of the Americas, disability adjusted life years are as high as 85%. Irrespective of the region [5], the largest burden of disability seems to be consistently concentrated among working-age individuals (19–69 years) [6]. The impact of NCDs on the economy is devastating and occurs through loss of human capital (in case of premature mortality), loss of worker productivity through absenteeism and presentism, premature retirements, healthcare expenditures, and the diversion of resources away from other sectors [7][8]. According to one estimate, NCDs’ global economic burden will amount to USD 47 trillion between 2010 and 2030 (base year 2010), or the equivalent of 75% of global gross domestic product [9][10].

According to GBD estimates for 2019, diets high in trans fatty acids (TFA) in the region of the Americas account for 108.9 thousand (10.2–146.3) deaths and for 2.1 million (0.2–2.7) DALYs. Processed and ultra-processed food and drink products (UPP) are the main dietary source of TFA, with their sales increasing at 3.1% a year in the Americas [11].

TFA are unsaturated fatty acids with at least one double bond in a trans position, which can be naturally produced in small quantities by ruminants, but most of the TFA in the modern diets are industrially produced. The technological properties of TFA in industrialized foods include longer shelf life, thermodynamic stability, and enhanced palatability of food products. Nevertheless, TFA intake has been associated with negative impacts cardiovascular health, through increasing low density lipoprotein cholesterol and decreasing high density lipoprotein cholesterol concentrations and causing systemic inflammation and endothelial dysfunction [12].

Industrially produced trans fatty acids (IP TFA) can be obtained through different technological processes such as the partial hydrogenation of vegetal and marine oils, the deodorization of vegetal and marine oils, deep frying at high temperature for long periods and the alkaline isomerization of linoleic acid [13][14][15]. Partially hydrogenated oils (PHO) are the main source of IP TFA and are created through the industrial hydrogenation of unsaturated oils into solid fats through the addition of hydrogen to unsaturated fatty acids under high temperature and high pressure in the presence of a metallic catalyst. Other industrial processes used in the refinement or production of vegetable oils, such as deodorization and full hydrogenation, also generate residual amounts of IP-TFA [16]. Finally, deep-frying at high temperature for long periods in industries or even at the household level can generate TFA especially when the vegetable oils used are rich in polyunsaturated fatty acids.

In response to this global public health issue, the World Health Organization has launched a technical action package to support countries, called REPLACE, which is based on the set of areas of action for supporting countries on the formulation, implementation, monitoring, and evaluation of IP TFA elimination policies and regulations [17].

Previous macro and microsimulation studies have proven the potential impact of TFA reduction on CVD mortality and may serve as important tools for advocacy and policy making, especially towards regulatory measures that limit the intake of IP TFA [18][19]. Nevertheless, none of these studies have been applied to Latin American or Low- and Middle-Income Countries to strengthen cost-effective policies in alignment with the REPLACE agenda.

## Methodology

### Research design

In this paper, we developed a comparative risk assessment model (the TFA Macrosimulation Model - TFAMM) and applied this tool to estimate the potential reductions in CVD mortality gained from the compared scenarios of TFA reduction/elimination, using Brazil as a case study.

### TFA consumption data

We used data relative to Brazil from the PHO and Non-PHO based Oils and Fats Market: Global Industry Analysis 2013–2017 and Forecast 2018–2026, produced in 2018 by Persistence Market Research for PAHO by request of the Pan American Health Organization [20]. The market data includes information on global, regional, and national PHO and non-PHO based oils and details on the market value, volume, and application (use). PHO is used in foods like bakery products, dairy and ice cream, chocolate and confectionery, breads, and cereals. National data on PHO use in foods can be used to estimate per capita percentage of total energy intake attributed to TFA, considering the population in the year of analysis and the estimated energy intake in the population according to dietary surveys or national food acquisition data (Supplementary Table S1).

### TFA macrosimulation model (TFAMM)

The TFA comparative risk assessment macrosimulation model estimates the potential CVD mortality reduction (coronary heart disease – CHD- and stroke) if trans-fat intake was reduced in diets. The primary outcome measure of this methodology is the total number of deaths prevented or postponed (DPP) that can be attributed to the reduction in this dietary risk factor. DPP are defined as the difference between the number of expected deaths in the year of analysis (age- and sex-specific CVD mortality) and the expected deaths if trans-fat intake was reduced in the diet of a specific population using Population Attributable Fraction (PAF).

The TFA macrosimulation model is a NCD scenario model that links a dietary risk factor (TFA intake) to mortality from NCD outcomes (cardiovascular diseases) and is methodologically similar to other comparative risk assessment methodologies, such as Global Burden of Disease [21] and the Preventable Risk Integrated Model (PRIME) [22]. It is based on the estimation of the change in the annual number of NCD deaths between baseline with current TFA consumption levels and alternate or counterfactual scenarios with low or no TFA intakes, along with uncertainty intervals. The model was created in Excel and has incorporated additional outputs related to other impacts of TFA reduction on DPP, such as Years of Life Lost, Years of Productive Life Lost, and related economic impacts of premature deaths.

The potential impact fractions (PIF) represent the proportional reduction in population disease or mortality would occur if exposure to a risk factor were reduced to an alternative exposure scenario. The estimated PIF for the mortality outcome (o) in each age group (a) and sex (s) stratum for each counterfactual scenario is represented by the following formula:

$${PIF}_{oas}= \frac{{\int }_{x=0}^{m}{RR}_{oa}\left(x\right){P}_{as}\left(x\right)dx- {\int }_{x=0}^{m}{RR}_{oa}\left(x\right){P}_{as}^{{\prime }}\left(x\right)dx}{{\int }_{x=0}^{m}{RR}_{oa}\left(x\right){P}_{as}\left(x\right)dx }$$

Where: *Pas(x)* and *P’as(x)* are the TFA intake distributions at the baseline and in the counterfactual scenario. *RRoa(x)* is the RR as a function of TFA intake in the energy of the diet, specific for outcome *(o)* and age.

Within the model, the averted number of deaths from CHD and stroke was computed by multiplying an age-, sex-, and cause-specific PAF by the current (pre-TFA policy) number of events for the same stratum.

Considering possible end-users of the model, it was developed to a user-friendly estimation of the potential impact of different policy scenarios for TFA reduction that can be applied to different national and regional contexts and that its outputs should be interpreted as the magnitude of change in attributable deaths from TFA consumption through policy implementation, using the Brazilian data as a proof-of-concept for the model.

### Description of the TFA macrosimulation model parameters

The TFA macrosimulation model requires to be parametrized or populated using three types of relevant baseline data for the population over 25 years of age. Context-specific and age- and sex-specific distribution of:

(i) The number of people living in the population;
(ii) Dietary risk factors (TFA intake and total energy in the diet);
(iii) The annual number of deaths from CVD (CHD and stroke) included in the model (Supplementary Table S2).

Common sources of these data include the most recent and available national census data for the number of people living in the population, national surveillance studies for energy intake and market research data for PHO use for food production (food and beverage industries, commercial and household) that capture the distribution of the TFA exposure and published meta-analyses of prospective epidemiological studies/cohort studies that estimate relative risks associated with TFA intake and NCD mortality. Together, these data reflect the current or the baseline situation of the TFA macrosimulation model.

The baseline data for the behavioral risk factors may then be modified, based on the knowledge of how the policy or the intervention is likely to affect them, to create different counterfactual scenarios. The creation of valid counterfactual scenarios, capturing the underlying mechanisms of change, may be informed by existing data or theory of change or a combination of the two.

The per capita consumption of TFA (g/day) was estimated based on the market research data available and conversion of the consumption to calories, assuming that 1 g of fat is equivalent to 9 kcal of energy. Then, the percentage of energy that comes from TFA is estimated using the dietary survey data for the country as the denominator.

In the model, TFA intake (% of total energy) was treated as a continuous risk exposure with a log-normal distribution in the population. The risk was assumed to be equivalent to that of meta-analyses of the observed association between TFA intake and CHD mortality, assuming a 2% energy replacement of TFAs with mono and polyunsaturated fatty acids (MUFAs and PUFAs), in prospective cohort studies and conservatively assumed that stroke mortality would be half of that observed for CHD.

The relative risks (including their confidence intervals) for CHD mortality per 2% of energy increase in TFA are 1.42 (1.28–1.57) for 25–34 years old, 1.40 (1.27–1.54) for 35–44 years old, 1.33 (1.22–1.45) for 45–54 years old, 1.27 (1.18–1.36) for 55–64 years old, and 1.22 (1.15–1.29) for 65–74 years old and 1.16 (1.11–1.21) for 75 + years old according to a meta-analysis by Wang et al. [12]. The estimations of RR for stroke mortality were estimated as half of the RR for CHD as used by Moreira et al. [23]. The relation between TFA intake and mortality was then separately parametrized for CHD and stroke assuming that TFA intake is a continuous risk factor and that health outcomes are mediated by a single RR parameter. The model considers a minimum theoretical risk of zero (0% intake of TFA represents RR = 0) and the distribution of RR according to modeled as intervals of 0.1% participation of TFA in the total energy in the diet from < 0.1% to > 2.4% is based on the formula:

$$R{R_i} = R{R^ \wedge }(x - y)/u$$

Where *(x)* is the midpoint for the TFA intake interval *(i)*, *(y)* is the midpoint for the first interval of RR distribution (in which RR = 0) and *(u)* is the unit increase according to the meta-analysis (Supplementary Tables S3 and S4).

### Costs of premature deaths

The estimated Years of Life Lost (YLL), which are part of the estimates for DALYs (Disability Adjusted Life Years), were calculated using the formula used by the GBD Study: YLL = N x L, where: N = the number of deaths from CVDs averted or postponed (estimated through the TFA macrosimulation model) and L = the standard life expectancy at the age of death in years for the Brazilian Population [24].

The Years of Productive Life Lost (YPLL) were estimated through the Human Capital Approach [25], which calculates the present value of potential time in the workforce (the measure of productivity) using country-specific data for 2017. YPLL was calculated by multiplying the YLL from age 15 to the pension age (60 years for women, and 65 years for men, in Brazil) by the average national wage and the labor force participation estimates from the Continuous National Household Sample Survey (PNAD), provided by IBGE [26].

### Development of counterfactual scenarios for Brazil

We designed three scenarios to model the effect of decreasing TFA consumption:

1. In Scenario A, we considered that TFA should not exceed 2% of total fat content for vegetable oils and soft spreadable margarines, and total TFA should not exceed more than 5% of fat content for all other foods. This was done to model the impact of the 2008 Trans Fat Free Americas declaration [27].
2. In Scenario B, we t limited industrially produced TFA to 2% of total fat content in all foods in the marketplace. This was done to model the impact of the TFA limits set in regulations from several countries [28].
3. In Scenario C, we considered the elimination of Partially Hydrogenated Oils (PHO) from the food market. This was done to model the effect of legislations banning PHOs, as they are considered the largest source of industrially produced TFA in most diets and assuming that PHOs will be replaced with mono and polyunsaturated fats [28].

For each scenario, the model generated the total numbers of deaths prevented or postponed as outputs. We present the results for Brazilian adults for 2018, rounded to 2 significant digits (uncertainty intervals, UI 95%, are not rounded).

The following modeling assumptions were considered for creating the counterfactual scenarios:

1. TFA sources from PHOs: We have assumed that food and beverage industries and food commerce (hotels, restaurants, and cafés) use mostly PHO fats and that most PHO oils are consumed at the household.
2. Mandatory or voluntary policies in place: Researchers will need to make assumptions about whether they would like to model a scenario in which the TFA reduction policy is implemented on a voluntary basis or on a mandatory basis. This would determine the policy reach, the extent to which the food industry reformulates their products and the extent of TFA reduction in the food market. PAHO recommends modelling a TFA policy implemented on a mandatory basis.
3. TFA intake threshold to be used: As there is no assumed lower limit for the safety of use of TFA, we have assumed that any level of consumption of TFA can be harmful and incorporated this assumption in the TFA model.
4. Estimating industry response to the TFA reduction or elimination policy: There may be several plausible responses of the food industry to the changes in TFA content in PHO and availability of PHO in the market. They may choose to eliminate all ingredients from PHOs or to comply to the limits that are enforced (i.e., in the 2% or 5% energy limits). As it is not possible to determine the distribution of industry responses (except in the PHO elimination scenario, which affects all the food market), we assumed that all food uses were equally affected by the limits in TFA content in PHOs.
5. Parameterization of the TFA model: Once the industry responses have been defined, researchers will have to implement the combined (or individual) changes on the consumption of TFA in the counterfactual scenarios. This will require a careful calibration of the inputs of the TFA model, by generating new age- and sex-specific estimates of mean total energy intake (kcal/day) a mean energy from TFA (mg/d).

We evaluated the robustness of our model through deterministic sensitivity analyses, by changing key model assumptions and inputs. We evaluated the impact of higher theoretical minimum risk exposure level for the contribution of TFA to total energy intake and higher relative risks for TFA intake RR for cardiovascular disease mortality (respectively, 15.4%±8.8% and 10.6%±6.3%) than estimated in the primary model.

### Probabilistic sensitivity analysis

Finally, the robustness of the model was assessed through sensitivity analyses, by changing key model assumptions and inputs. We evaluated the impact of higher minimum theoretical risks for TFA intake and CVD outcomes (0.2%). Lastly, we explored lower and higher RR for TFA intake and CVD outcomes (10% differences) than estimated in the primary model.

### Uncertainty analysis

The uncertainty analysis (Monte Carlo) was incorporated in the model to calculate probabilistic 95% uncertainty intervals (95% UI) for all model outputs, based on 5,000 draws from specified probabilistic distributions for the model input variables (the prevalence of exposure and the relative risks between exposure and health outcomes), using the Erastz add-on [29]. This also allows the model to incorporate the usual random error (sampling error) in the Relative Risks (RR) and exposure prevalence as well as other potential sources of uncertainty such as uncontrolled confounding or extrapolation from a source to a target population, because of the assumption of the portability of the RRs from the metanalyses. In case of the modeling uses in this study, the final population attributable fractions (PAF) are based on the weighted sum of the PAF for each exposure, sex, and age-group strata. Again, after repeated draws and repeated calculations of the PAF, Monte Carlo limits can be obtained [30]. The parameter distributions used for the input variables to the DPP calculations and intermediate estimations are shown in the online supplementary material (Supplementary Tables S2 to S7).

## Results

In 2018, the per capita use of PHO was estimated at 5.8 g/day, in Brazil. The impact of TFA reduction policy scenarios show significant differences on TFA intake. In Brazil, the scenario with 2% limit in TFA for oils and fats and 5% for other food products would reduce TFA intake by 14–16% per year in 2018, while the overall limit of 2% would represent a 54.6% reduction in TFA intake. Banning PHO would virtually eliminate industrially produced TFA from PHO to zero (Table 1).

**Table 1:** Trans fatty acid (TFA) and partially hydrogenated oils (PHO) use for food production and estimates of TFA per capita intake in 2018, Brazil

Variable	Estimates
**Daily per capita PHO consumption (g/day)**	5.8
**Daily per capita PHOs for household use (g/day)**	2.5
**Average per capita TFA from PHO (g/day)**	**1.2**
% of energy from TFA	0.68%
**Estimated per capita participation of TFA in total energy intake**	
**Scenario 1**	0.57%
**Scenario 2**	0.31%
**Scenario 3**	0.00%

In scenario 1 applied to mortality data from 2018, the 2% limit for oils and fats and 5% limit of TFAs for other foods could avert or postpone approximately 2,000 deaths (UI 95% (1,899-2,142) from cardiovascular disease among Brazilian adults, representing the reduction of 14.5 thousand Years of Life Lost. Premature deaths represented 47% of these deaths in 2018, that yearly could represent less 4.1 thousand Years of Productive Life Years and US$ 32.1 million savings in productivity losses to the economy.

In scenario 2, also applied to 2018, considering the 2% limit of TFA in all food products, we estimated approximately 6,300 prevented or postponed deaths (UI 95% 5,925-6,684) and 45 thousand Years of Life Lost, prevented. The reduction in TFA intake could result in saving 12,6 thousand years of productive life and reduce losses in economic productivity equivalent to US$ 100.2 million.

Finally, in scenario 3, in which the banning of PHO was modelled, we estimated that approximately 10,500 deaths could be prevented or postponed (UI 95% 9,963 − 10,909), which correspond to reducing 75.5 thousand Years of Life Lost, in 2018. The prevention or postponement of premature deaths, which have a significant impact on workforce productivity, could reduce Years of Productive Life Lost by 21.1 thousand years, corresponding to US$ 166.7 million in savings to the economy because of premature deaths (Tables 2 and 3).

Regarding age and sex patterns, the total deaths averted among men and women were almost equally distributed and shown no statistical difference for both CHD and for stroke (Supplementary Tables S10-S12), while productivity losses due to premature death increase with age and peaked at 50 to 54 years among men and at 45 to 49 years among women. Over 60% of the productivity losses for both sexes were concentrated within the 30 to 50 years of age range, which represents a large burden to the economy, considering the impact on the labor force of the country.

**Table 2:** Deaths prevented or postponed among adults and their uncertainty intervals (95%) by sex in each TFA reduction scenario in Brazil, 2018

		Number of deaths prevented or postponed		
		**Coronary heart disease**	**Stroke**	**Total**
		**Mean (95% UI)**	**Mean (95% UI)**	**Mean (95% UI)**
**Scenario 1**	Male Female Total	600 (605–683) 700 (635–715) 1,300 (1,240-1,398)	400 (337–382) 300 (322–363) 700 (679–704)	1,000 (942-1,064) 1,000 (957-1,078) 2,000 (1,899-2,142)
**Scenario 2**	Male Female Total	2,000 (1,887-2,219) 2,100 (1,981-2,232) 4,100 (3,869-4,361)	1,100 (1,052 − 1,191) 1,100 (1,004 − 1,132) 2,200 (5,998-6,557)	3,100 (2,939-3,321) 3,200 (3,049 − 3,363) 6,300 (5,925-6,684)
**Scenario 3**	Male Female Total	3,300 (3,140-3,544) 3,500 (3,297-3,713) 6,800 (6,437-7,257)	1,900 (1,750–1981) 1,800 (1,671-1,867) 3,700 (3,525-3,652)	5,200 (4,890-5,525) 5,300 (5,073 − 5,384) 10,500 (9,963 − 10,909)

**Table 3:** Savings in losses of productivity in each TFA reduction scenario by sex and age-group in Brazil, 2018

	*Savings (in thousand US$)*		
	***Scenario 1*** **Mean(95% UI)**	***Scenario 2*** **Mean (95% UI)**	***Scenario 3*** **Mean (95% UI)**
***Male***			
***25–29 years***	785.4 (708 to 868.4)	2,450.2 (2,208.6 to 2,709)	4,078.2 (3,676.1 to 4,509)
***30–34 years***	1,383.2 (1,246.8 to 1,529.3)	4,315.3 (3,889.8 to 4,771.1)	7,182.9 (6,474.7 to 7,941.7)
***35–39 years***	2,243.7 (2,035.4 to 2,468.1)	6,999.7 (6,349.7 to 7,699.7)	11,650.9 (10,569 to 12,816.0)
***40–44 years***	3,321.5 (3,013.1 to 3,653.7)	10,362.2 (9,400.0 to 11,398.4)	17,247.9 (15,646.3 to 18,972.7)
***45–49 years***	3,539.9 (3,247.1 to 3,859.3)	11,043.4 (10,130.0 to 12,039.8)	18,378.8 (16,858.7 to 20,037.0)
***50–54 years***	3,949.1 (3,622.5 to 4,305.4)	12,320.0 (11,301.1 to 13,431.6)	20,503.4 (18,807.6 to 22,353.3)
***55–59 years***	2,769 (2,572.8 to 2,965.2)	8638.5 (8026.3 to 9250.7)	14,374.4 (13,355.7 to 15,393.1)
***60–64 years***	1233.4 (1,146.0 to 1,320.8)	3,848.0 (3,575.3 to 4,120.7)	6403 (5,949.2 to 6,856.8)
***Subtotal***	19,225.1 (18,066.2 to 20,410.5)	59,977.2 (56,361.9 to 63,675.4)	99,819.5 (93,802.5 to 105,974.4)
***Female***			
***25–29 years***	857.9 (801.7 to 889.8)	2,676.3 (2,500.9 to 2,775.7)	4,455.2 (4,163.2 to 4,620.7)
***30–34 years***	1,588.2 (1,484.1 to 1,647.2)	4,954.7 (4,630.0 to 5,138.8)	8,247.9 (7,707.4 to 8,554.3)
***35–39 years***	2,176.7 (2,042.3 to 2,253.6)	6,790.9 (6,371.7 to 7,030.9)	11,303.9 (10,606.1 to 11,703.5)
***40–44 years***	2,656.2 (2,492.2 to 2,750.1)	8,286.8 (7,775.3 to 8,579.7)	13,793.9 (12,942.4 to 14,281.5)
***45–49 years***	2,683.4 (2,537.3 to 2,767.0)	8,371.5 (7,915.6 to 8,632.4)	13,932.5 (13,173.8 to 14,366.7)
***50–54 years***	2,180.2 (2,061.5 to 2,248.1)	6,801.7 (6,431.3 to 7,013.7)	11,319.9 (10,703.4 to 11,672.6)
***55–59 years***	747.0 (712.2 to 763.7)	2,330.5 (2,221.9 to 2,382.4)	3,878.0 (3,697.4 to 3,964.4)
***Subtotal***	12,889.7 (12,519.7 to 13,0740)	40,212.6 (39,058.4 to 40,787.7)	66,931.1 (65,010.0 to 67,888.3)
***Total***	32,114.9 (30,524.0 to 33,422.5)	100,189.9 (95,226.8 to 104,269.3)	166,750.7 (158,490.4 to 173,540.3)

### Sensitivity analyses

Considering the different sensitivity analysis scenarios, the modelled estimates of CVD events attributable to the consumption of TFA varied from − 7% (lower RR) to + 17% (higher RR) compared to the primary model estimate. The other sensitivity analysis scenario (higher theoretical minimum exposure level) had relatively minor impact on the modeled estimates compared to the primary model (Supplementary Tables S8 and S9).

## Discussion

This study provides a detailed description of an innovative and easy-to-use tool for modeling the impact of different policy scenarios to limit the intake of industrially-produced trans fatty acids, to inform policy and decision-making and strengthen advocacy towards TFA regulatory measures that, are in alignment with the WHO REPLACE agenda and is applicable to specific country contexts, as for Brazil.

The application of the TFA modeling tool in this study shows that, despite the reduction in the use of TFA by food industries over the last decade, the burden of cardiovascular disease associated to TFA intake is still relevant. Considering the policy scenarios for reducing TFA intake, 2% TFA limits to oils and fats and 5% limits of TFA to other food products (counterfactual scenario 1) would have lesser impacts than setting a 2% limit to all products (counterfactual scenario 2), which would reduce per capita TFA intake to half of the first scenario. The largest impact may be obtained from banning PHO (counterfactual scenario 3), considering that they are a major source of TFA in the diet and could prevent of postpone over 10 thousand deaths/year compared to keeping the current TFA intake.

The economic savings associated to premature deaths are also maximized when PHOs are eliminated from the food market and represent a five-fold reduction in productivity losses compared to the less stringent TFA reduction scenario. These counterfactual scenarios that were analyzed highlight the need for more improvement in regulatory measures to significantly reduce the exposure of populations to TFA intake and its consequences on cardiovascular health considering the multiple sources of PHO and TFA in the diets.

Considering an effectiveness hierarchy of TFA reduction policies, multicomponent interventions including a legislative ban on products appear the most effective strategy to reduce TFA intake. On the other hand, downstream interventions targeting individuals in domestic or work settings appear consistently less effective [31].

The estimates of impact of our study are consistent with other modelling studies in TFA reduction in countries as Argentina [32], Australia [33], the UK [34][35] as well as with data produced by the Global Burden of Disease project for Brazil [6], which use similar underlying methods and assumptions. Also, the results confirm that the decision taken by Brazilian authorities to ban industrially produced TFA from foods was the best from a policy effectiveness approach, compared to other policy options such as limiting TFA content in foods [36].

Policy interventions to remove industrial TFAs from foods are the most effective public health approach for reducing TFA intake and decreasing the burden of noncommunicable diseases. Several countries proved the feasibility of such policies during the last decades. For example, in Denmark, TFA intake was progressively reduced through multicomponent interventions and supported by strong political will over a decade, followed by a legislative ban that virtually eliminated TFAs in margarines and vegetable shortenings. Most other countries only have achieved voluntary TFAs limits, reflecting concerns about political feasibility and generally lower levels of public pressure for change [31].

In the region of the Americas, voluntary approaches to product reformulation have been attempted but had limited effectiveness when compared to regulatory approaches. Although nine countries reported developing some policy related to TFA to this moment, few have implemented and/or enforced these measures appropriately.

Regulatory agencies in several countries in the Americas required the food industry to declare the amount of trans fat in food on the Nutrition Facts label for over one decade. For example, in the Mercosul region trans-fat declaration was made mandatory in nutritional labels in 2003, followed by the Food and Drug Administration (FDA) of the United States, in 2006. Subsequently, in November 2013, the FDA made a preliminary determination that PHOs are not “generally recognized as safe” (GRAS) for use in food, confirmed in 2015 [37], opening the policy settings for interventions aimed to eliminating TFA in the region. Consequently, member States of the Pan American Health Organization (PAHO) approved during the 57th session of the Directing Council, the Plan of Action of the elimination of industrially produced trans fatty acids to achieve elimination by 2023.

Reducing the TFA involves multiple components, yet regulations aimed to reducing the content of TFA in foods or eliminating PHOs are among the most cost-effective policies. For example, in Brazil, TFA consumption has been gradually reduced over the last decades, likely influenced by the mandatory declaration of trans fats in packaged foods and the implementation of trans fat free claims in 2003, and the voluntary commitment of national food industries to the Declaration of Rio de Janeiro, in 2008, which encouraged food reformulation in many food categories [27][38].

Nevertheless, because of the limited impact of food reformulation induced by labeling policies and voluntary reductions [39], the PHOs have continued to be used by small food industries, food services and in the households, so the Brazilian National Health Surveillance Agency (Anvisa) approved a new regulation that firstly sets a 2% limit to TFAs in the fat content of foods and subsequently eliminates the use of PHO in the food market by 2023 [40].

Despite the choice for the most impactful policy alternatives for TFA reduction in Brazil, the long deadline for PHO elimination in the country can represent the occurrence of thousands of preventable deaths from cardiovascular diseases until full compliance to the regulations. Therefore, policy choices must seriously consider the timeframe of regulations prioritizing health impacts over other factors and among the alternatives for TFA reduction, as, in Brazil, PHO elimination may achieve three to five times more deaths averted and costs saved compared to setting limits to TFA content in foods.

### Strengths and limitations

The limitations of the TFA model assumptions need to be highlighted. Because the TFA consumption estimates are produced from market research information instead of dietary survey data, the per capita values are the same regardless of sex or age-group. Nevertheless, they can be adjusted by sex- and age-specific total energy averages from dietary surveys. Consequently, it is likely that the impact of TFA reduction will be underestimated, especially for younger people, who generally consume more TFA in their diets. Also, as the percentage of use of PHO for food production is not available by country, we assume that the percentage for the entire region of the Americas will be applicable to each country.

Also, because per capita intakes of TFA were estimated equally for men and women and different age-groups, it is likely that the results represent conservative estimates of exposure for groups that are likely to consume more energy and TFA food sources, such as young men.

The consumption estimates, when generated from PHO and non-PHO producers, is likely to be more updated and complete than dietary surveys because it includes PHOs for all foods and preparations and are based on historical market data, while food composition tables may not capture the current food reformulation and the different uses of PHO according to the size or type of food business or household use.

This TFA macrosimulation model, similarly to other comparative risk assessment models, is relatively straightforward to use and adaptable to different settings. Since the required data are largely based on population-level estimates of current risk factor distributions and disease-specific mortalities, they are likely to be collected in most countries. However, a drawback of this simplicity is that the health outcomes estimated by the model (deaths delayed or averted) do not allow for temporal considerations of the health impact and does not consider lag times between changes in risk exposure and health outcomes neither the lifetime exposure to risk factors. Therefore, the model cannot be used to estimate the effect of risk factor scenarios on standard epidemiological measures such as disability or quality adjusted life years or on measures that incorporate morbidity such as health-related quality of life.

Additionally, the set of assumptions identified in this study are not exhaustive and there may be other assumptions, not included, that could be considered when using this TFA macrosimulation model to estimate changes in NCD mortality. For example, the model does not take account for the possible interactions and competing risks considering CHD and stroke as model outcomes because it would require the models to have data on the joint distribution of risk factors within the population of interest for the baseline scenarios, which are not available in the literature. Consequently, we assume an absence of interaction terms in our model in order to generate conservative estimates of health effects of the modeled scenarios.

The burden of disease on years of life lost to premature death is part of the disability-adjusted life years, therefore the reduction of TFA intake on DALYs is much higher. Similarly, regarding the economic impacts of the scenarios on productivity represent only part of the total outcomes (premature deaths), but other aspects such as presentism and absenteeism are not covered by the model. Given the characteristics of the data sources and the conservative assumptions underpinning the estimates for groups with higher exposure to dietary risk factors decreases in the model, the overall effects in mortality seen are likely to be underestimated.

## Conclusion

This study shows that our TFA macrosimulation model can efficiently compare different policy scenarios for trans fats reduction policies at the country level and the results suggest that the elimination of PHOs from the food market in Brazil that will be in effect by 2023 may help prevent a substantial number of deaths from CVDs and significantly reduce the health burden of trans fatty acids in the country. Additionally, this trans fats macrosimulation model can be a useful public health tool to support policymakers in implementing regulations for TFA reduction and elimination in other countries.

## Electronic supplementary material

Below is the link to the electronic supplementary material.

Supplementary Material 1

## Data Availability

All relevant data on the model are within the paper and the model is available in the GitHub repository (https://github.com/eduardonilson/TFA-CVD-model/tree/main). Microdata and aggregated data for Population, Workforce, and Household Budget Survey for Brazil are publicly accessible through the Brazilian Institute of Geography and Statistics (https://www.ibge.gov.br/), and the mortality data is also publicly available through the Brazilian National Health System’s Department of Informatics (http://tabnet.datasus.gov.br/).
