# Supplementary material for: Development and application of the TFA macrosimulation model: a case study of modelling the impact of trans fatty acid (TFA) elimination policies in Brazil
Source: BMC Public Health. 2022 Nov 2;22:2010. doi: 10.1186/s12889-022-14361-9 (PMC9632015; doi:10.1186/s12889-022-14361-9)
Supplement: Supplementary file 1 — Supplementary Material 1 [file 12889_2022_14361_MOESM1_ESM.docx]

**Supplementary Materials**

**Development and application of the TFA Macrosimulation Model: a case study of modelling the impact of Trans Fatty Acid (TFA) elimination policies in Brazil**

**BMC Public Health**

**Eduardo Augusto Fernandes Nilson, Neha Khandpur, Fabio da Silva Gomes**

**The authors have provided this Appendix to give readers additional information about their work.**

**Summary of evidence about the risks or trans fatty acids (TFA) intake**

***The morbidity and mortality associated with NCDs remain unacceptably high in the Americas.***

Noncommunicable diseases (NCDs) are the leading cause of global mortality globally, affecting more people each year than all other causes combined and dietary risk factors represent the largest burden among the modifiable NCD risk factors (1). According to the 2019 Global Burden of Disease Study, non-communicable diseases (NCDs) account for 73.4% (72.5 - 74.3%) of global mortality, or 42.0 million (40,1 – 43,9) deaths and 1,62 billion (1.43-1.81) Disability-Adjusted Life Years (DALYs) (2). Premature mortality from NCDs results in over 15 million deaths of people between the ages of 30 and 69 years, more than 85% of whom live in low- and middle-income countries (3).

In 2019, cardiovascular diseases (CVD) such as ischemic heart disease and stroke, the most common causes of NCD mortality, were responsible for an estimated 15.8 million deaths and 349.5 million DALYs (4). In some regions of the Americas, disability adjusted life years are as high as 85%. Irrespective of the region (5), the largest burden of disability seems to be consistently concentrated among working-age individuals (19 – 69 years) (6). The impact of NCDs on the economy is devastating and occurs through loss of human capital (in case of premature mortality), loss of worker productivity through absenteeism and presentism, premature retirements, healthcare expenditures, and the diversion of resources away from other sectors (7)(8). According to one estimate, NCDs’ global economic burden will amount to USD 47 trillion between 2010–2030 (base year 2010), or the equivalent of 75% of global gross domestic product (9)(10).

Trans fatty acids are unsaturated fatty acids with at least one double bond in a trans position. They are naturally produced in small quantities in the stomachs of ruminants, such as cows and sheep, but are more commonly found in the diet from partially hydrogenated vegetable oils in processed foods. Trans fatty acids are frequently used by food industries because they allow longer shelf life, thermodynamic stability, and enhanced palatability of food products. TFA increase low density lipoprotein cholesterol concentrations, decrease high density lipoprotein cholesterol concentrations, and can also cause systemic inflammation as well as endothelial dysfunction (11).

Industrially produced trans fatty acids (IP TFA) can be obtained through different technological processes such as the partial hydrogenation of vegetal and marine oils, the deodorization of vegetal and marine oils, deep frying at high temperature for long periods and the alkaline isomerization of linoleic acid (12)(13)(14).

Partially hydrogenated oils (PHO) are the main source of IP TFA and are created through the industrial hydrogenation of unsaturated oils into solid fats through the addition of hydrogen to unsaturated fatty acids under high temperature and high pressure in the presence of a metallic catalyst. Other industrial processes used in the refinement or production of vegetable oils, such as deodorization and full hydrogenation, also generate residual amounts of IP-TFA (15) . Finally, deep-frying at high temperature for long periods in industries or even at the household level can generate TFA especially when the vegetable oils used are rich in polyunsaturated fatty acids.

According to GBD estimates for 2019, diets high in TFA in the region of the Americas account for 108.9 thousand (10.2 - 146.3) deaths and for 2.1 million (0.2 - 2.7) DALYs. Processed and ultra-processed food and drink products (UPP) are the main dietary source of TFA and their sales increasing at 3.1% a year in the Americas (12).

***Eliminating industrially produced trans fats from food supply is a cost-effective intervention to prevent CVDs***

Considering an effectiveness hierarchy of TFA reduction policies, multicomponent interventions including a legislative ban on products appear the most effective strategy to reduce TFA intake. On the other hand, downstream interventions targeting individuals in domestic or work settings appear consistently less effective (13).

Policy interventions to remove industrial TFAs from foods have are suggested as the most effective public health approach for reducing TFA intake and decreasing the burden of noncommunicable diseases. Several countries proved the feasibility of such policies during the last decades. For example, in Denmark, TFA intake was progressively reduced through multicomponent interventions and supported by strong political will over a decade, followed by a legislative ban that virtually eliminated TFAs in margarines and vegetable shortenings. Most other countries only have achieved voluntary TFAs limits, reflecting concerns about political feasibility and generally lower levels of public pressure for change (13).

In the region of the Americas, voluntary approaches to product reformulation have been attempted but had limited effectiveness when compared to regulatory approaches. Although nine countries reported developing some policy related to TFA to this moment, few have implemented and/or enforced these measures appropriately.

Regulatory agencies in several countries in the Americas required the food industry to declare the amount of trans fat in food on the Nutrition Facts label for over one decade. For example, in the Mercosul region trans-fat declaration was made mandatory in nutritional labels in 2003, followed by the Food and Drug Administration (FDA) of the United States, in 2006. Subsequently, in November 2013, the FDA made a preliminary determination that PHOs are not “generally recognized as safe” (GRAS) for use in food, confirmed in 2015 (14), opening the policy settings for interventions aimed to eliminating TFA in the region.

Consequently, member States of the Pan American Health Organization (PAHO) approved during the 57th session of the Directing Council, the Plan of Action of the elimination of industrially-produced trans fatty acids to achieve elimination by 2023.

In Brazil, the mandatory declaration of trans fats in packaged foods and the implementation of trans fat free claims in 2003, along with the voluntary commitment of national food industries to the Declaration of Rio de Janeiro, in 2008, has incentive the reduction of trans fat content in many food categories through food reformulation. Nevertheless, PHOs have continued to be used by small food industries, food services and in the households.

***Evidence that demonstrates the impact of the elimination of TFA is crucial to informing regulatory measures***

Nevertheless, countries lack the necessary technical support to implement recommended interventions under WHO’s policy package, (REPLACE) (15), and, within this background, research protocols and impact estimates are needed to help advance the development and finalization of tools and resources requested by Member States to support the TFA elimination policies.

Previous macro and microsimulation studies (16)(17) have proven the potential impact of TFA reduction on CVD mortality and may serve as important tools for advocacy and policy making, especially towards regulatory measures that limit the intake of industrially-produced trans fatty acids.

The TFA Macrosimulation Model was developed as a tool that could help estimate the potential reductions in CVD mortality gained from the compared scenarios of TFA reduction/elimination. It is a food policy macrosimulation tool that links uses current epidemiological knowledge to estimate the effectiveness of public health interventions which alter dietary risk factors such as the intake of TFA, saturated fats, sodium, and sugars.

**TFA consumption data**

We used data from the PHO and Non-PHO based Oils and Fats Market: Global Industry Analysis 2013-2017 and Forecast 2018-2026, produced in 2018 by Persistence Market Research for PAHO (18).

The market data includes information on global, regional, and national PHO and non-PHO based oils and details on the market value, volume, and application (use). Regarding the uses of PHO, the use in foods includes categories such as food and beverages industry (bakery, dairy and ice cream, chocolate and confectionery, breads and cereals and other uses), commercial (hotels, restaurants, and cafés) and household applications.

National data on PHO use in foods was used to estimate per capita percentage of total energy intake attributed to TFA, considering the population in the year of the analysis (if possible, >1 year of age or alternatively with the entire population) and the estimated energy intake in the population according to dietary surveys or national food acquisition data.

**The TFA macrosimulation model**

The TFA macrosimulation methodology estimates the potential age- and sex-specific CVD mortality reduction (coronary heart disease – CHD- and stroke) if trans-fat intake was reduced in diets.

We have chosen to create multiple counterfactual scenarios that account for different levels of changes in TFA intake in the populations and compare them to the baseline scenario to estimate population attributable fractions and changes in CVD mortality for each TFA reduction or elimination policy.

We designed three scenarios to model the effect of decreasing TFA consumption:

1. In Scenario A, we considered 2% limits of TFA in oils and fats and 5% limits in other food products considering the energy contribution of TFAs in foods.

2. In Scenario B, we considered a 2% energy limit to all products considering the energy contribution of TFAs in foods.

3. In Scenario C, we considered the elimination of Partially Hydrogenated Oils (PHO) from the food market.

**Description of the TFA macrosimulation model parameters**

The TFA macrosimulation model requires to be parametrized or populated using three types of relevant baseline data. Context-specific and age- and sex-specific distribution of:

(i) The number of people living in the population;

(ii) Dietary risk factors (TFA intake and total energy in the diet);

(iii) The annual number of deaths from CVD (coronary heart diseases and cerebrovascular diseases) included in the model.

Population data from Brazil was based on the most recent and available national census data for the number of people living in the population. The average energy of the diets of the adult population was obtained from recent national dietary surveys and the TFA and PHO consumption was captured from market research data. Published meta-analyses of prospective epidemiological studies/cohort studies estimate relative risks associated to TFA intake and NCD mortality and CVD mortality was obtained from the official national health systems. Together, these data reflect the current or the baseline situation of the TFA macrosimulation model for both countries.

The TFA macrosimulation model is a NCD scenario model that links a dietary risk factor (TFA intake) to mortality from NCD outcomes(cardiovascular diseases) and is methodologically similar to the GBD methodologies, such as the Preventable Risk Integrated Model (PRIME) (19) and other modelling studies in the literature (17). It is based on the estimation of the change in the annual number of NCD deaths between the baseline and counterfactual scenarios, along with uncertainty intervals, calculated based on 5000 iterations of a Monte Carlo analysis. The model was created in Excel and has incorporated additional outputs related to other impacts of TFA reduction, such as Years of Life Lost, Years of Productive Life Lost, and related economic impacts of premature deaths.

In the model, TFA intake (% of total energy) was treated as a continuous risk exposure with a log-normal distribution in the population. The risk was assumed to be equivalent to that of meta-analyses of the observed association between TFA intake and CHD mortality in prospective cohort studies and conservatively assuming that stroke mortality would be half of that observed for CHD. The relative risks (including their uncertainty intervals) for CHD mortality per 2% of energy increase in TFA are 1.42 (1.28–1.57) for 25–34 years old, 1.40 (1.27–1.54) for 35–44 years old, 1.33 (1.22–1.45) for 45–54 years old, 1.27 (1.18–1.36) for 55–64 years old, and 1.22 (1.15–1.29) for 65–74 years old and 1.16 (1.11–1.21) for 75+ years old (20). The relation between TFA intake and CHD and stroke mortality was then parametrized and modeled as intervals of 0.1% participation of TFA in total energy, from <0.1% to >2.4% of the total energy in the diet.

The uncertainty analysis (Monte Carlo) was incorporated in the model to calculate probabilistic 95% uncertainty intervals (95% UI) for all model outputs, based on 5,000 draws from specified probabilistic distributions for the model input variables. This also allows the model to incorporate the usual random error (sampling error) in the RR and exposure prevalence as well as other potential sources of uncertainty such as uncontrolled confounding or extrapolation from a source to a target population, because of the assumption of the portability of the RRs from the metanalyses. In case of the modeling uses in this study, the final potential impact fractions (PIF) are based on the weighted sum of the PIF for each exposure, sex and age-group strata. Again, after repeated draws and repeated calculations of the AF, Monte Carlo limits can be obtained (21). The parameter distributions used for the input variables to the DPP calculations are shown in the online supplementary material.

Additional analyses of the burden of premature deaths include the estimated Years of Life Lost (YLL), which are part of the estimates for DALYs (Disability Adjusted Life Years), were calculated using the formula used by GBD: YLL = N x L, where: N = the number of deaths from CVDs averted or postponed (estimated through the TFA model) and L = the standard life expectancy at the age of death in years for the population. And the Years of Productive Life Lost (YPLL) were estimated through the Human Capital Approach (22), which calculates the present value of potential time in the workforce (the measure of productivity) using country-specific data for the year of analysis. YPLL was calculated by multiplying the YLL from age 15 to the pension age by the average national wage and the labor force participation estimates.

**Modeling assumptions for creating the counterfactual scenario**

A modeling exercise that follows these phases would need to make certain assumptions to inform the calibration of the TFA model. These assumptions would also guide the identification of appropriate empirical data sources, or theoretical models, particularly for Phase 1. They include:

1. TFA sources from PHOs: We have assumed that food and beverage industries and food commerce (hotels, restaurants, and cafés) use mostly PHO fats and that most PHO oils are consumed at the household. According to data from Brazil, the TFA content of PHOs in 2018 varied from 2% to 40%, with an estimated average of 21% (23).

2. Mandatory or voluntary policies in place: We assumed that TFA policy in all scenarios was implemented on a mandatory basis, as recommended by PAHO.

3. TFA intake threshold to be used: As there is no assumed lower limit for the safety of use of TFA, we have assumed that any level of consumption of TFA can be harmful and incorporated this assumption in the TFA model.

4. Estimating industry response to the TFA reduction or elimination policy: There may be several plausible responses of the food industry to the changes in TFA content in PHO and availability of PHO in the market. They may choose to eliminate all ingredients from PHOs or to comply to the limits that are enforced (ie, in the 2% or 5% energy limits). As it is not possible to determine the distribution of industry responses (except in the PHO elimination scenario, which affects all the food market), we assumed that all food uses were equally affected by the limits in TFA content in PHOs.

5. Parameterization of the TFA model: Once the industry responses have been defined, researchers will have to implement the combined (or individual) changes on the consumption of TFA in the counterfactual scenarios. This will require a careful calibration of the inputs of the TFA model, by generating new age- and sex-specific estimates of mean total energy intake (kcal/day) a mean energy from TFA (mg/d).

**The per capita TFA estimation strengths and limitations**

Because the TFA consumption estimates are produced from market research information instead of dietary survey data, the per capita values are the same regardless of sex or age-group. Nevertheless, they can be adjusted by sex- and age-specific total energy averages from dietary surveys. Consequently, it is likely that the impact of TFA reduction will be underestimated, especially for younger people, who generally consume more TFA in their diets. Also, as the percentage of use of PHO for food production is not available by country, we assume that the percentage for the entire region of the Americas will be applicable to each country.

Also, because per capita intakes of TFA were estimated equally for men and women and different age-groups, it is likely that the results represent conservative estimates of exposure for groups that are likely to consume more energy and TFA food sources, such as young men.

The consumption estimates, when generated from PHO and non-PHO producers, is likely to be more updated and complete than dietary surveys because it includes PHOs for all foods and preparations and are based on historical market data, while food composition tables may not capture the current food reformulation and the different uses of PHO according to the size or type of food business or household use.

**The TFA macrosimulation model limitations**

The TFA macrosimulation model, similarly to PRIME, is relatively straight forward to use and adaptable to different settings, as long as the data required to parametrize the model are available for the context in question. Since the required data are largely based on population-level estimates of current risk factor distributions and disease-specific mortalities, they are likely to be collected in multiple settings. However, a drawback of this simplicity is that the health outcomes estimated by the model (deaths delayed or averted) do not allow for temporal considerations of the health impact and therefore the model cannot be used to estimate the effect of risk factor scenarios on standard epidemiological measures such as disability or quality adjusted life years or on measures that incorporate morbidity such as health-related quality of life.

The macrosimulation model also does not account for the possible interactions between behavioral risk factors for NCDs and is unable to incorporate the effect of time lag between exposure and disease outcome. Therefore, the model does not consider lifetime exposure to risk factors when calculating population attributable fractions and was not designed to predict the future but rather to estimate the difference between two possible scenarios. It is also worth mentioning that the set of assumptions identified in this report are not exhaustive and there may be other assumptions, not included, that could be considered when using this TFA macrosimulation model to estimate changes in NCD mortality.

The burden of disease on years of life lost to premature death is part of the disability-adjusted life years, therefore the reduction of TFA intake on DALYs is much higher. Similarly, regarding the economic impacts of the scenarios on productivity represent only part of the total outcomes (premature deaths), but other aspects such as presentism and absenteeism are not covered by the model.

**References**

1. Afshin A, Sur PJ, Fay KA, Cornaby L, Ferrara G, Salama JS, et al. Health effects of dietary risks in 195 countries, 1990–2017: a systematic analysis for the Global Burden of Disease Study 2017. Lancet [Internet]. 2019;393(19):1958–72. Available from: https://doi.org/10.1016/S0140-6736(19)30041-8

2. Roth GA, Abate D, Abate KH, Abay SM, Abbafati C, Abbasi N, et al. Global, regional, and national age-sex-specific mortality for 282 causes of death in 195 countries and territories, 1980–2017: a systematic analysis for the Global Burden of Disease Study 2017. Lancet [Internet]. 2018;392(10159):1736–88. Available from: https://doi.org/10.1016/s0140-6736(18)32203-7

3. World Health Organization (WHO). Noncommunicable diseases [Internet]. 2018. Available from: https://www.who.int/news-room/fact-sheets/detail/noncommunicable-diseases

4. Institute for Health Metrics and Evaluation (IHME). Global burden of disease 2019 [Internet]. 2020. Available from: https://vizhub.healthdata.org/gbd-compare

5. Chen S, Kuhn M, Prettner K, Bloom DE. The macroeconomic burden of noncommunicable diseases in the United States: Estimates and projections. PLoS One [Internet]. 2018;13(11):e0206702. Available from: https://doi.org/10.1371/journal.pone.0206702

6. IHME. Global Health Data Exchange - GHDx [Internet]. 2019. Available from: http://ghdx.healthdata.org/gbd-results-tool

7. Bertram MY, Sweeny K, Lauer JA, Chisholm D, Sheehan P, Rasmussen B, et al. Investing in non-communicable diseases: an estimation of the return on investment for prevention and treatment services. Lancet [Internet]. 2018;391(10134):2071–8. Available from: http://dx.doi.org/10.1016/S0140-6736(18)30665-2

8. Rasmussen B, Sweeny K, Sheehan P. Health and Economy: The Impact of Wellness on Workforce Productivity in Global Markets [Internet]. 2016. Available from: https://www.uschamber.com/sites/default/files/documents/files/global_initiative_on_health_and_the_economy_-_report.pdf

9. Bloom DE, Cafiero ET, Jané-Llopis E, Abrahams-Gessel S, Bloom LR, Fathima S, et al. PGDA Working Paper No. 87 - The Global Economic Burden of Noncommunicable Diseases [Internet]. Geneva, Switzerland; 2012. (Program on the Global Demography of Aging). Report No.: 87. Available from: https://cdn1.sph.harvard.edu/wp-content/uploads/sites/1288/2013/10/PGDA_WP_87.pdf

10. Bloom DE, Chen S, Kuhn M, McGovern ME, Oxley L, Prettner K. The economic burden of chronic diseases: Estimates and projections for China, Japan, and South Korea. J Econ Ageing [Internet]. 2019;13(11):e0206702. Available from: https://doi.org/10.1016/j.jeoa.2018.09.002

11. Wang Q, Afshin A, Yakoob MY, Singh GM, Rehm CD, Khatibzadeh S, et al. Impact of nonoptimal intakes of saturated, polyunsaturated, and trans fat on global burdens of coronary heart disease. J Am Heart Assoc. 2016;5(1):e002891.

12. Pan American Health Organization. Ultra-processed food and drink products in Latin America: Sales, sources, nutrient profiles, and policy implications [Internet]. Washington, DC: PAHO - Pan American Health Organization; 2019. 72 p. Available from: https://iris.paho.org/handle/10665.2/51094

13. Hyseni L, Bromley H, Kypridemos C, O’Flaherty M, Lloyd-Williams F, Guzman-Castillo M, et al. Systematic review of dietary trans-fat reduction interventions. Bull World Health Organ [Internet]. 2017;95:821–30. Available from: https://dx.doi.org/10.2471/BLT.16.189795

14. Food and Drug Administration. Tentative Determination Regarding Partially Hydrogenated Oils; Request for Comments and for Scientific Data and Information [Internet]. 2013. p. 67169–75. Available from: https://www.govinfo.gov/content/pkg/FR-2013-11-08/pdf/2013-26854.pdf

15. Ghebreyesus TA, Frieden TR. REPLACE: a roadmap to make the world trans fat free by 2023. Lancet [Internet]. 2018;391(10134):P1978-1980. Available from: https://doi.org/10.1016/S0140-6736(18)31083-3

16. Allen K, Pearson-Stuttard J, Hooton W, Diggle P, Capewell S, O’Flaherty M. Potential of trans fats policies to reduce socioeconomic inequalities in mortality from coronary heart disease in England: Cost effectiveness modelling study. BMJ [Internet]. 2015;351:h4583. Available from: https://doi.org/10.1136/bmj.h4583

17. Wu JHY, Zheng M, Catterall E, Downs S, Thomas B, Veerman L, et al. Contribution of trans-fatty acid intake to coronary heart disease burden in Australia: A modelling study. Nutrients [Internet]. 2017;9(1):77. Available from: https://dx.doi.org/10.3390%2Fnu9010077

18. Persistence Market Research. PHO and Non-PHO based Oils and Fats Market: Global Industry Analysis 2013-2017 and Forecast 2018-2026. 2018.

19. Scarborough P, Harrington RA, Mizdrak A, Zhou LM, Doherty A. The Preventable Risk Integrated ModEl and Its Use to Estimate the Health Impact of Public Health Policy Scenarios. Scientifica (Cairo). 2014;2014:748750.

20. Wang Q, Afshin A, Yakoob MY, Singh GM, Rehm CD, Khatibzadeh S, et al. Impact of Nonoptimal Intakes of Saturated, Polyunsaturated, and Trans Fat on Global Burdens of Coronary Heart Disease. J Am Heart Assoc [Internet]. 2016;5(1):e002891. Available from: https://doi.org/10.1161/jaha.115.002891

21. Steenland K, Armstrong B. An overview of methods for calculating the burden of disease due to specific risk factors. Epidemiology [Internet]. 2006;17(5):512–9. Available from: https://doi.org/10.1097/01.ede.0000229155.05644.43

22. Zhang W, Bansback N, Anis AH. Measuring and valuing productivity loss due to poor health: A critical review. Soc Sci Med [Internet]. 2011;72(2):185–92. Available from: https://doi.org/10.1016/j.socscimed.2010.10.026

23. Anvisa. Relatório de Análise de Impacto Regulatório sobre Ácidos Graxos Trans em Alimentos. 2019.

24. IBGE. Brazilian Population Estimates [Internet]. 2017. Available from: https://www.ibge.gov.br/en/statistics/social/population/18448-population-estimates.html?=&t=o-que-e

25. Ministério da Saúde. SIM - Mortality Information System [Internet]. 2017. Available from: http://tabnet.datasus.gov.br/cgi/deftohtm.exe?sim/cnv/obt10uf.def

26. IBGE. Pesquisa de orçamentos familiares 2017-2018 : análise do consumo alimentar pessoal no Brasil [Internet]. Rio de Janeiro, Brazil; 2020. 120 p. Available from: https://biblioteca.ibge.gov.br/visualizacao/livros/liv101742.pdf

**Table S0. TFA Macrosimulation Model input parameters to estimate the attributable deaths to FA intake in Brazil.**

| **Model inputs** | **BMI (RR per 5 units)** | **Source** |
| --- | --- | --- |
| *Baseline characteristics* |  |  |
| Population count (by age and sex) |  | Brazilian Population Estimates (IBGE) |
| Total diet energy |  | Brazilian National Household Budget Survey (POF 2017-2018/IBGE) |
| Industrial TFA by use |  | Persistence Market Research (2018) |
| Deaths |  | Mortality Information System - SIM 2019 |
| Relative risks for coronary heart disease | 35-59y: 1,50 (1,39–1,62)  60–69 y: 1,40 (1,32-1,49)  70–79 y: 1,31 (1,23-1,40)  80-89y: 1,30 (1,17-1,45) | Wang et al (2016) |
| Relative risks for stroke | 35-59y: 1,76 (1,52-2,04)  60–69 y: 1,49 (1,34-1,67)  70–79 y: 1,33 (1,19-1,48)  80-89y: 1,10 (0,94–1,30) | Wang et al (2016) |

**Table S1 – TFA and PHO use for food production and estimates of TFA per capita intake from 2013 to 2018 in Brazil.**

| **Year** | **2018** |
| --- | --- |
| **Annual volume of PHO in the Americas (tons)** | 494,948 |
| **Proportion of the volume of PHO destined to the food chain in the Americas** | 0.8734 |
| **Estimated annual volume of PHO commercialized for foods in the Americas (tons)** | **432,287** |
| **Proportion of the volume destined to household use** | 0.3803 |
| **Annual volume of PHO destined to household uses in the Americas (tons)** | **188,219** |
| **Estimated population (>1 year of age)** | **204,798,011** |
| **Annual per capita PHO consumption (g/year)** | 2,111 |
| **Daily per capita PHO consumption (g/day)** | 5.8 |
| **Annual per capita PHOs for household use (g/year)** | 919 |
| **Daily per capita PHOs for household use (g/day)** | 2.5 |
| **Maximum per capita TFA from PHO (g/day) - 40% TFA in PHOs** | **2.3** |
| **Average per capita TFA from PHO (g/day) - 21% TFA in PHOs** | **1.2** |
| % of energy from TFA | 0.67901% |
| Total energy (kcal/day) | 1609.68 |
| **Estimated per capita participation of TFA in total energy intake** |  |
| **Scenario 1** | 0.57% |
| **Scenario 2** | 0.31% |
| **Scenario 3** | 0.00% |

**Table S2. Total population, deaths from coronary heart disease and stroke and average energy participation of trans fatty acids among Brazilian adults aged 25 years and more (2018).**

|  |  | **Deaths** | | | | **Baseline** | |
| --- | --- | --- | --- | --- | --- | --- | --- |
|  | **Population** | **Coronary heart disease** | | **Stroke** | | **TFA intake (%)** | **SD TFA** |
| **Male** | | |  | |  |  |  |
| **25-29y** | 8,634,062 | 306 | | 192 | | 0.68 | 0.22 |
| **30-34y** | 8,816,350 | 599 | | 322 | | 0.68 | 0.22 |
| **35-39y** | 7,879,629 | 961 | | 563 | | 0.68 | 0.22 |
| **40-44y** | 6,882,229 | 1,760 | | 990 | | 0.68 | 0.22 |
| **45-49y** | 6,266,088 | 3,088 | | 1,577 | | 0.68 | 0.22 |
| **50-54y** | 5,659,602 | 4,955 | | 2,496 | | 0.68 | 0.22 |
| **55-59y** | 4,678,702 | 6,624 | | 3,370 | | 0.68 | 0.22 |
| **60-64y** | 3,655,025 | 7,864 | | 4,651 | | 0.68 | 0.22 |
| **65-69y** | 2,672,043 | 8,663 | | 5,776 | | 0.68 | 0.22 |
| **70-74y** | 1,793,543 | 8,180 | | 6,596 | | 0.68 | 0.22 |
| **75-79y** | 1,222,286 | 8,017 | | 7,547 | | 0.68 | 0.22 |
| **80y+** | 1,246,775 | 13,764 | | 15,790 | | 0.68 | 0.22 |
|  |  |  | |  | |  |  |
| **Female** | | |  | |  |  |  |
| **25-29y** | 8,542,758 | 81 | | 149 | | 0.68 | 0.22 |
| **30-34y** | 8,821,067 | 199 | | 326 | | 0.68 | 0.22 |
| **35-39y** | 7,976,620 | 443 | | 573 | | 0.68 | 0.22 |
| **40-44y** | 7,062,001 | 816 | | 899 | | 0.68 | 0.22 |
| **45-49y** | 6,536,311 | 1.360 | | 1.522 | | 0.68 | 0.22 |
| **50-54y** | 6,027,703 | 2.107 | | 2.061 | | 0.68 | 0.22 |
| **55-59y** | 5,120,890 | 3.089 | | 2.578 | | 0.68 | 0.22 |
| **60-64y** | 4,141,984 | 4.051 | | 3.283 | | 0.68 | 0.22 |
| **65-69y** | 3,172,657 | 4.983 | | 4.243 | | 0.68 | 0.22 |
| **70-74y** | 2,283,021 | 5.635 | | 5.350 | | 0.68 | 0.22 |
| **75-79y** | 1,691,282 | 6.636 | | 7.116 | | 0.68 | 0.22 |
| **80y+** | 2,062,198 | 17.104 | | 21.898 | | 0.68 | 0.22 |

**Table S3. Relative risks for death from coronary heart disease estimated according to the energy participation of trans fatty acids in the diet (%) by age-group.**

|  | **CHD mortality per 2% increase in TFA** | **0%** | **0.2%** | **0.4%** | **0.6%** | **0.8%** | **1.0%** | **1.2%** | **1.4%** | **1.6%** | **1.8%** | **2.0%** | **2.2%** | **2.4%** |
| --- | --- | --- | --- | --- | --- | --- | --- | --- | --- | --- | --- | --- | --- | --- |
| **25-29y** | 1.00 | 1.00 | 1.00 | 1.00 | 1.00 | 1.00 | 1.00 | 1.00 | 1.00 | 1.00 | 1.00 | 1.00 | 1.00 | 1.00 |
| **30-34y** | 1.00 | 1.00 | 1.00 | 1.00 | 1.00 | 1.00 | 1.00 | 1.00 | 1.00 | 1.00 | 1.00 | 1.00 | 1.00 | 1.00 |
| **35-39y** | 1.42 | 1.00 | 1.04 | 1.07 | 1.11 | 1.15 | 1.19 | 1.24 | 1.28 | 1.33 | 1.37 | 1.42 | 1.47 | 1.53 |
| **40-44y** | 1.42 | 1.00 | 1.04 | 1.07 | 1.11 | 1.15 | 1.19 | 1.24 | 1.28 | 1.33 | 1.37 | 1.42 | 1.47 | 1.53 |
| **45-49y** | 1.40 | 1.00 | 1.03 | 1.07 | 1.11 | 1.14 | 1.18 | 1.22 | 1.27 | 1.31 | 1.36 | 1.40 | 1.45 | 1.50 |
| **50-54y** | 1.40 | 1.00 | 1.03 | 1.07 | 1.11 | 1.14 | 1.18 | 1.22 | 1.27 | 1.31 | 1.36 | 1.40 | 1.45 | 1.50 |
| **55-59y** | 1.33 | 1.00 | 1.03 | 1.06 | 1.09 | 1.12 | 1.15 | 1.19 | 1.22 | 1.26 | 1.29 | 1.33 | 1.37 | 1.41 |
| **60-64y** | 1.33 | 1.00 | 1.03 | 1.06 | 1.09 | 1.12 | 1.15 | 1.19 | 1.22 | 1.26 | 1.29 | 1.33 | 1.37 | 1.41 |
| **65-69y** | 1.27 | 1.00 | 1.02 | 1.05 | 1.07 | 1.10 | 1.13 | 1.15 | 1.18 | 1.21 | 1.24 | 1.27 | 1.30 | 1.33 |
| **70-74y** | 1.27 | 1.00 | 1.02 | 1.05 | 1.07 | 1.10 | 1.13 | 1.15 | 1.18 | 1.21 | 1.24 | 1.27 | 1.30 | 1.33 |
| **75-79y** | 1.22 | 1.00 | 1.02 | 1.04 | 1.06 | 1.08 | 1.10 | 1.13 | 1.15 | 1.17 | 1.20 | 1.22 | 1.24 | 1.27 |
| **80y+** | 1.22 | 1.00 | 1.02 | 1.04 | 1.06 | 1.08 | 1.10 | 1.13 | 1.15 | 1.17 | 1.20 | 1.22 | 1.24 | 1.27 |

**Table S4. Relative risks for death from stroke estimated according to the energy participation of trans fatty acids in the diet (%) by age-group.**

|  | **Stroke mortality per 2% increase in TFA** | **0%** | **0.2%** | **0.4%** | **0.6%** | **0.8%** | **1.0%** | **1.2%** | **1.4%** | **1.6%** | **1.8%** | **2.0%** | **2.2%** | **2.4%** |
| --- | --- | --- | --- | --- | --- | --- | --- | --- | --- | --- | --- | --- | --- | --- |
| **25-29y** | 1.21 | 1.00 | 1.02 | 1.04 | 1.06 | 1.08 | 1.10 | 1.12 | 1.14 | 1.17 | 1.19 | 1.21 | 1.23 | 1.26 |
| **30-34y** | 1.21 | 1.00 | 1.02 | 1.04 | 1.06 | 1.08 | 1.10 | 1.12 | 1.14 | 1.17 | 1.19 | 1.21 | 1.23 | 1.26 |
| **35-39y** | 1.20 | 1.00 | 1.02 | 1.04 | 1.06 | 1.08 | 1.10 | 1.12 | 1.14 | 1.16 | 1.18 | 1.20 | 1.22 | 1.25 |
| **40-44y** | 1.20 | 1.00 | 1.02 | 1.04 | 1.06 | 1.08 | 1.10 | 1.12 | 1.14 | 1.16 | 1.18 | 1.20 | 1.22 | 1.25 |
| **45-49y** | 1.17 | 1.00 | 1.02 | 1.03 | 1.05 | 1.06 | 1.08 | 1.10 | 1.11 | 1.13 | 1.15 | 1.17 | 1.18 | 1.20 |
| **50-54y** | 1.17 | 1.00 | 1.02 | 1.03 | 1.05 | 1.06 | 1.08 | 1.10 | 1.11 | 1.13 | 1.15 | 1.17 | 1.18 | 1.20 |
| **55-59y** | 1.14 | 1.00 | 1.01 | 1.03 | 1.04 | 1.05 | 1.07 | 1.08 | 1.09 | 1.11 | 1.12 | 1.14 | 1.15 | 1.16 |
| **60-64y** | 1.14 | 1.00 | 1.01 | 1.03 | 1.04 | 1.05 | 1.07 | 1.08 | 1.09 | 1.11 | 1.12 | 1.14 | 1.15 | 1.16 |
| **65-69y** | 1.11 | 1.00 | 1.01 | 1.02 | 1.03 | 1.04 | 1.05 | 1.06 | 1.08 | 1.09 | 1.10 | 1.11 | 1.12 | 1.13 |
| **70-74y** | 1.11 | 1.00 | 1.01 | 1.02 | 1.03 | 1.04 | 1.05 | 1.06 | 1.08 | 1.09 | 1.10 | 1.11 | 1.12 | 1.13 |
| **75-79y** | 1.08 | 1.00 | 1.01 | 1.02 | 1.02 | 1.03 | 1.04 | 1.05 | 1.06 | 1.06 | 1.07 | 1.08 | 1.09 | 1.10 |
| **80y+** | 1.08 | 1.00 | 1.01 | 1.02 | 1.02 | 1.03 | 1.04 | 1.05 | 1.06 | 1.06 | 1.07 | 1.08 | 1.09 | 1.10 |

**Table S5. Prevalence of exposure (TFA intake) at baseline according to the energy participation of trans fatty acids in the diet (%) by age-group.**

|  | **TFA in the total energy of the diet** | | | | | | | | | | | | |
| --- | --- | --- | --- | --- | --- | --- | --- | --- | --- | --- | --- | --- | --- |
| **Age** | **0%** | **0.2%** | **0.4%** | **0.6%** | **0.8%** | **1.0%** | **1.2%** | **1.4%** | **1.6%** | **1.8%** | **2.0%** | **2.2%** | **2.4%** |
| **25-29y** | 0.00000 | 0.00686 | 0.14303 | 0.19497 | 0.10293 | 0.03691 | 0.01119 | 0.00316 | 0.00088 | 0.00024 | 0.00007 | 0.00002 | 0.00001 |
| **30-34y** | 0.00000 | 0.00686 | 0.14303 | 0.19497 | 0.10293 | 0.03691 | 0.01119 | 0.00316 | 0.00088 | 0.00024 | 0.00007 | 0.00002 | 0.00001 |
| **35-39y** | 0.00000 | 0.00686 | 0.14303 | 0.19497 | 0.10293 | 0.03691 | 0.01119 | 0.00316 | 0.00088 | 0.00024 | 0.00007 | 0.00002 | 0.00001 |
| **40-44y** | 0.00000 | 0.00686 | 0.14303 | 0.19497 | 0.10293 | 0.03691 | 0.01119 | 0.00316 | 0.00088 | 0.00024 | 0.00007 | 0.00002 | 0.00001 |
| **45-49y** | 0.00000 | 0.00686 | 0.14303 | 0.19497 | 0.10293 | 0.03691 | 0.01119 | 0.00316 | 0.00088 | 0.00024 | 0.00007 | 0.00002 | 0.00001 |
| **50-54y** | 0.00000 | 0.00686 | 0.14303 | 0.19497 | 0.10293 | 0.03691 | 0.01119 | 0.00316 | 0.00088 | 0.00024 | 0.00007 | 0.00002 | 0.00001 |
| **55-59y** | 0.00000 | 0.00686 | 0.14303 | 0.19497 | 0.10293 | 0.03691 | 0.01119 | 0.00316 | 0.00088 | 0.00024 | 0.00007 | 0.00002 | 0.00001 |
| **60-64y** | 0.00000 | 0.00686 | 0.14303 | 0.19497 | 0.10293 | 0.03691 | 0.01119 | 0.00316 | 0.00088 | 0.00024 | 0.00007 | 0.00002 | 0.00001 |
| **65-69y** | 0.00000 | 0.00686 | 0.14303 | 0.19497 | 0.10293 | 0.03691 | 0.01119 | 0.00316 | 0.00088 | 0.00024 | 0.00007 | 0.00002 | 0.00001 |
| **70-74y** | 0.00000 | 0.00686 | 0.14303 | 0.19497 | 0.10293 | 0.03691 | 0.01119 | 0.00316 | 0.00088 | 0.00024 | 0.00007 | 0.00002 | 0.00001 |
| **75-79y** | 0.00000 | 0.00686 | 0.14303 | 0.19497 | 0.10293 | 0.03691 | 0.01119 | 0.00316 | 0.00088 | 0.00024 | 0.00007 | 0.00002 | 0.00001 |
| **80y+** | 0.00000 | 0.00686 | 0.14303 | 0.19497 | 0.10293 | 0.03691 | 0.01119 | 0.00316 | 0.00088 | 0.00024 | 0.00007 | 0.00002 | 0.00001 |

**Table S6. PIF for CHD deaths associated with TFA intake by age-group in counterfactual scenario 3.**

|  | **TFA in the total energy of the diet** | | | | | | | | | | | | |  |
| --- | --- | --- | --- | --- | --- | --- | --- | --- | --- | --- | --- | --- | --- | --- |
| **Age** | **0%** | **0.2%** | **0.4%** | **0.6%** | **0.8%** | **1.0%** | **1.2%** | **1.4%** | **1.6%** | **1.8%** | **2.0%** | **2.2%** | **2.4%** | **TOTAL** |
| **25-29y** | 0.000% | 0.000% | 0.000% | 0.000% | 0.000% | 0.000% | 0.000% | 0.000% | 0.000% | 0.000% | 0.000% | 0.000% | 0.000% | 0.000% |
| **30-34y** | 0.000% | 0.000% | 0.000% | 0.000% | 0.000% | 0.000% | 0.000% | 0.000% | 0.000% | 0.000% | 0.000% | 0.000% | 0.000% | 0.000% |
| **35-39y** | 0.000% | 0.025% | 1.032% | 2.124% | 1.532% | 0.705% | 0.262% | 0.088% | 0.028% | 0.009% | 0.003% | 0.001% | 0.001% | 5.810% |
| **40-44y** | 0.000% | 0.025% | 1.032% | 2.124% | 1.532% | 0.705% | 0.262% | 0.088% | 0.028% | 0.009% | 0.003% | 0.001% | 0.001% | 5.810% |
| **45-49y** | 0.000% | 0.024% | 0.989% | 2.036% | 1.467% | 0.674% | 0.251% | 0.084% | 0.027% | 0.009% | 0.003% | 0.001% | 0.001% | 5.565% |
| **50-54y** | 0.000% | 0.024% | 0.989% | 2.036% | 1.467% | 0.674% | 0.251% | 0.084% | 0.027% | 0.009% | 0.003% | 0.001% | 0.001% | 5.565% |
| **55-59y** | 0.000% | 0.020% | 0.836% | 1.719% | 1.234% | 0.565% | 0.209% | 0.070% | 0.023% | 0.007% | 0.002% | 0.001% | 0.001% | 4.688% |
| **60-64y** | 0.000% | 0.020% | 0.836% | 1.719% | 1.234% | 0.565% | 0.209% | 0.070% | 0.023% | 0.007% | 0.002% | 0.001% | 0.001% | 4.688% |
| **65-69y** | 0.000% | 0.017% | 0.695% | 1.429% | 1.022% | 0.466% | 0.172% | 0.058% | 0.018% | 0.006% | 0.002% | 0.001% | 0.000% | 3.886% |
| **70-74y** | 0.000% | 0.017% | 0.695% | 1.429% | 1.022% | 0.466% | 0.172% | 0.058% | 0.018% | 0.006% | 0.002% | 0.001% | 0.000% | 3.886% |
| **75-79y** | 0.000% | 0.014% | 0.577% | 1.184% | 0.845% | 0.384% | 0.142% | 0.047% | 0.015% | 0.005% | 0.002% | 0.000% | 0.000% | 3.215% |
| **80y+** | 0.000% | 0.014% | 0.577% | 1.184% | 0.845% | 0.384% | 0.142% | 0.047% | 0.015% | 0.005% | 0.002% | 0.000% | 0.000% | 3.215% |

**Table S7. PIF for stroke deaths associated with TFA intake by age-group in counterfactual scenario 3.**

|  | **TFA in the total energy of the diet** | | | | | | | | | | | | |  |
| --- | --- | --- | --- | --- | --- | --- | --- | --- | --- | --- | --- | --- | --- | --- |
| **Age** | **0%** | **0.2%** | **0.4%** | **0.6%** | **0.8%** | **1.0%** | **1.2%** | **1.4%** | **1.6%** | **1.8%** | **2.0%** | **2.2%** | **2.4%** |  |
| **25-29y** | 0,000% | 0,013% | 0,555% | 1,139% | 0,812% | 0,369% | 0,136% | 0,045% | 0,014% | 0,005% | 0,001% | 0,000% | 0,000% | 6,176% |
| **30-34y** | 0,000% | 0,013% | 0,555% | 1,139% | 0,812% | 0,369% | 0,136% | 0,045% | 0,014% | 0,005% | 0,001% | 0,000% | 0,000% | 6,176% |
| **35-39y** | 0,000% | 0,013% | 0,530% | 1,088% | 0,776% | 0,352% | 0,130% | 0,043% | 0,014% | 0,004% | 0,001% | 0,000% | 0,000% | 5,901% |
| **40-44y** | 0,000% | 0,013% | 0,530% | 1,088% | 0,776% | 0,352% | 0,130% | 0,043% | 0,014% | 0,004% | 0,001% | 0,000% | 0,000% | 5,901% |
| **45-49y** | 0,000% | 0,011% | 0,444% | 0,910% | 0,647% | 0,293% | 0,108% | 0,036% | 0,011% | 0,004% | 0,001% | 0,000% | 0,000% | 4,928% |
| **50-54y** | 0,000% | 0,011% | 0,444% | 0,910% | 0,647% | 0,293% | 0,108% | 0,036% | 0,011% | 0,004% | 0,001% | 0,000% | 0,000% | 4,928% |
| **55-59y** | 0,000% | 0,009% | 0,366% | 0,749% | 0,532% | 0,241% | 0,088% | 0,029% | 0,009% | 0,003% | 0,001% | 0,000% | 0,000% | 4,052% |
| **60-64y** | 0,000% | 0,009% | 0,366% | 0,749% | 0,532% | 0,241% | 0,088% | 0,029% | 0,009% | 0,003% | 0,001% | 0,000% | 0,000% | 4,052% |
| **65-69y** | 0,000% | 0,007% | 0,301% | 0,616% | 0,437% | 0,197% | 0,072% | 0,024% | 0,008% | 0,002% | 0,001% | 0,000% | 0,000% | 3,329% |
| **70-74y** | 0,000% | 0,007% | 0,301% | 0,616% | 0,437% | 0,197% | 0,072% | 0,024% | 0,008% | 0,002% | 0,001% | 0,000% | 0,000% | 3,329% |
| **75-79y** | 0,000% | 0,005% | 0,221% | 0,453% | 0,321% | 0,145% | 0,053% | 0,018% | 0,006% | 0,002% | 0,001% | 0,000% | 0,000% | 2,446% |
| **80y+** | 0,000% | 0,005% | 0,221% | 0,453% | 0,321% | 0,145% | 0,053% | 0,018% | 0,006% | 0,002% | 0,001% | 0,000% | 0,000% | 2,446% |

**Table S8. Probabilistic sensitivity analysis: deaths prevented or postponed (DPP) from cardiovascular diseases (CVD), considering 10% lower and higher relative risks (RR).**

|  | **10% lower RR** | | |  |  |  | **10% higher RR** | | |
| --- | --- | --- | --- | --- | --- | --- | --- | --- | --- |
|  | **CVD DPP** | **UI 95%** | |  |  |  | **CVD DPP** | **UI 95%** | |
| **Male** |  |  |  |  |  | **Male** |  |  |  |
| **25-29y** | 49 | 44 | 54 |  |  | **25-29y** | 56 | 50 | 62 |
| **30-34y** | 92 | 83 | 102 |  |  | **30-34y** | 105 | 95 | 117 |
| **35-39y** | 143 | 130 | 158 |  |  | **35-39y** | 165 | 149 | 181 |
| **40-44y** | 260 | 236 | 286 |  |  | **40-44y** | 299 | 271 | 329 |
| **45-49y** | 366 | 336 | 399 |  |  | **45-49y** | 429 | 394 | 468 |
| **50-54y** | 585 | 537 | 638 |  |  | **50-54y** | 687 | 630 | 749 |
| **55-59y** | 634 | 589 | 678 |  |  | **55-59y** | 764 | 710 | 818 |
| **60-64y** | 777 | 722 | 832 |  |  | **60-64y** | 937 | 870 | 1,003 |
| **65-69y** | 706 | 665 | 746 |  |  | **65-69y** | 880 | 830 | 931 |
| **70-74y** | 701 | 661 | 742 |  |  | **70-74y** | 875 | 825 | 925 |
| **75-79y** | 505 | 483 | 527 |  |  | **75-79y** | 675 | 646 | 704 |
| **80y+** | 928 | 888 | 968 |  |  | **80y+** | 1,240 | 1,187 | 1,294 |
| **Female** |  |  |  |  |  | **Female** |  |  |  |
| **25-29y** | 19 | 17 | 21 |  |  | **25-29y** | 22 | 19 | 24 |
| **30-34y** | 44 | 40 | 49 |  |  | **30-34y** | 50 | 45 | 56 |
| **35-39y** | 84 | 77 | 93 |  |  | **35-39y** | 97 | 88 | 107 |
| **40-44y** | 146 | 133 | 161 |  |  | **40-44y** | 168 | 152 | 185 |
| **45-49y** | 200 | 184 | 218 |  |  | **45-49y** | 235 | 216 | 256 |
| **50-54y** | 296 | 272 | 323 |  |  | **50-54y** | 348 | 319 | 379 |
| **55-59y** | 334 | 310 | 358 |  |  | **55-59y** | 403 | 374 | 431 |
| **60-64y** | 434 | 403 | 465 |  |  | **60-64y** | 524 | 486 | 561 |
| **65-69y** | 434 | 409 | 459 |  |  | **65-69y** | 542 | 510 | 573 |
| **70-74y** | 508 | 479 | 537 |  |  | **70-74y** | 634 | 597 | 670 |
| **75-79y** | 437 | 418 | 455 |  |  | **75-79y** | 584 | 559 | 609 |
| **80y+** | 1,202 | 1,150 | 1,253 |  |  | **80y+** | 1,607 | 1,538 | 1,676 |
|  |  |  |  |  |  |  |  |  |  |
| **Total** | **9,885** | **9,265** | **10,523** |  |  | **Total** | **12,324** | **11,561** | **13,106** |

**Table S9. Probabilistic sensitivity analysis: deaths prevented or postponed (DPP) from cardiovascular diseases (CVD), considering a theoretical minimum risk exposure of 0.2% of TFA in the total energy of the diet.**

|  | **CVD DPP** | **UI 95%** | |  |
| --- | --- | --- | --- | --- |
| **Male** |  |  |  |  |
| **25-29y** | 47 | 37 | 58 |  |
| **30-34y** | 89 | 69 | 108 |  |
| **35-39y** | 140 | 110 | 171 |  |
| **40-44y** | 255 | 199 | 308 |  |
| **45-49y** | 366 | 282 | 450 |  |
| **50-54y** | 585 | 456 | 718 |  |
| **55-59y** | 651 | 503 | 786 |  |
| **60-64y** | 803 | 630 | 972 |  |
| **65-69y** | 746 | 587 | 908 |  |
| **70-74y** | 744 | 577 | 900 |  |
| **75-79y** | 564 | 445 | 686 |  |
| **80y+** | 1.042 | 818 | 1.259 |  |
| **Female** |  |  |  |  |
| **25-29y** | 19 | 14 | 23 |  |
| **30-34y** | 43 | 33 | 53 |  |
| **35-39y** | 83 | 65 | 101 |  |
| **40-44y** | 144 | 113 | 175 |  |
| **45-49y** | 202 | 157 | 248 |  |
| **50-54y** | 298 | 232 | 365 |  |
| **55-59y** | 345 | 268 | 421 |  |
| **60-64y** | 445 | 351 | 546 |  |
| **65-69y** | 463 | 362 | 560 |  |
| **70-74y** | 538 | 420 | 656 |  |
| **75-79y** | 490 | 382 | 595 |  |
| **80y+** | 1.349 | 1.064 | 1.615 |  |
|  |  |  |  |  |
| **Total** | **10,453** | 8.176 | 12.680 |  |

**Table S10. Hypothesis test for evaluating the difference in PIF (population impact fractions) between males and females for Scenario 1 (2% limit for TFA in oils and 5% limit for other food products).**

| *Coronary heart disease* | *Male* | *Female* |
| --- | --- | --- |
| Mean | 0.043642 | 0.043355 |
| Known variance | 0.000545 | 0.000537 |
| Observations | 14 | 14 |
| Pearson correlation | 0.999796 |  |
| Hipothesized mean diferences | 0 |  |
| gl | 13 |  |
| Stat t | 2.126612 |  |
| P(T<=t) one-tail | 0.026588 |  |
| t Critical one-tail | 1.770933 |  |
| P(T<=t) two-tail | 0.053177 |  |
| t Critical two-tail | 2.160369 |  |
|  |  |  |
|  |  |  |
| *Stroke* | *Male* | *Female* |
| Mean | 0.022699 | 0.022722 |
| Known variance | 0.00015 | 0.000151 |
| Observations | 14 | 14 |
| Pearson correlation | 0.999883 |  |
| Hipothesized mean diferences | 0 |  |
| Gl | 13 |  |
| Stat t | -0.44478 |  |
| P(T<=t) one-tail | 0.331896 |  |
| t Critical one-tail | 1.770933 |  |
| P(T<=t) two-tail | 0.663792 |  |
| t Critical two-tail | 2.160369 |  |

**Table S11. Hypothesis test for evaluating the difference in PIF (population impact fractions) between males and females for Scenario 2 (2% limit for TFA in all food products).**

| *Coronary heart disease* | *Male* | *Female* |
| --- | --- | --- |
| Mean | 0.013165 | 0.013156 |
| Known variance | 5.08E-05 | 4.86E-05 |
| Observations | 14 | 14 |
| Pearson correlation | 0.997116 |  |
| Hipothesized mean diferences | 0 |  |
| gl | 13 |  |
| Stat t | 0.061335 |  |
| P(T<=t) one-tail | 0.476013 |  |
| t Critical one-tail | 1.770933 |  |
| P(T<=t) two-tail | 0.952026 |  |
| t Critical two-tail | 2.160369 |  |
|  |  |  |
|  |  |  |
| *Stroke* | *Male* | *Female* |
| Mean | 0.00677 | 0.00677 |
| Known variance | 1.35E-05 | 1.35E-05 |
| Observations | 14 | 14 |
| Pearson correlation | 0.999791 |  |
| Hipothesized mean diferences | 0 |  |
| Gl | 13 |  |
| Stat t | 0.266023 |  |
| P(T<=t) one-tail | 0.397197 |  |
| t Critical one-tail | 1.770933 |  |
| P(T<=t) two-tail | 0.794394 |  |
| t Critical two-tail | 2.160369 |  |

**Table S12. Hypothesis test for evaluating the difference in PIF (population impact fractions) between males and females for Scenario 3 (elimination of partially hydrogenated oils).**

| *Coronary heart disease* | *Male* | *Female* |
| --- | --- | --- |
| Mean | 0.072916 | 0.072901 |
| Known variance | 0.001508 | 0.001502 |
| Observations | 14 | 14 |
| Pearson correlation | 0.999962 |  |
| Hipothesized mean diferences | 0 |  |
| gl | 13 |  |
| Stat t | 0.157073 |  |
| P(T<=t) one-tail | 0.4388 |  |
| t Critical one-tail | 1.770933 |  |
| P(T<=t) two-tail | 0.877601 |  |
| t Critical two-tail | 2.160369 |  |
|  |  |  |
|  |  |  |
| *Stroke* | *Male* | *Female* |
| Mean | 0.038265 | 0.038288 |
| Known variance | 0.000428 | 0.000427 |
| Observations | 14 | 14 |
| Pearson correlation | 0.99997 |  |
| Hipothesized mean diferences | 0 |  |
| Gl | 13 |  |
| Stat t | -0.53535 |  |
| P(T<=t) one-tail | 0.300724 |  |
| t Critical one-tail | 1.770933 |  |
| P(T<=t) two-tail | 0.601448 |  |
| t Critical two-tail | 2.160369 |  |
